# CpG island density and its correlations with genomic features in mammalian genomes

**DOI:** 10.1186/gb-2008-9-5-r79

**Published:** 2008-05-13

**Authors:** Leng Han, Bing Su, Wen-Hsiung Li, Zhongming Zhao

**Affiliations:** 1 Department of Psychiatry, Virginia Commonwealth University, Richmond, VA 23298, USA; 2 State Key Laboratory of Genetic Resources and Evolution, Kunming Institute of Zoology, Chinese Academy of Sciences, Kunming, Yunnan 650223, China; 3 Graduate School, Chinese Academy of Sciences, Beijing 100039, China; 4 Kunming Primate Research Center, Chinese Academy of Sciences, Kunming, Yunnan 650223, China; 5 Department of Ecology and Evolution, University of Chicago, Chicago, IL 60637, USA; 6 Department of Human Genetics and Center for the Study of Biological Complexity, Virginia Commonwealth University, Richmond, VA 23284, USA

## Abstract

**Background:**

CpG islands, which are clusters of CpG dinucleotides in GC-rich regions, are considered gene markers and represent an important feature of mammalian genomes. Previous studies of CpG islands have largely been on specific loci or within one genome. To date, there seems to be no comparative analysis of CpG islands and their density at the DNA sequence level among mammalian genomes and of their correlations with other genome features.

**Results:**

In this study, we performed a systematic analysis of CpG islands in ten mammalian genomes. We found that both the number of CpG islands and their density vary greatly among genomes, though many of these genomes encode similar numbers of genes. We observed significant correlations between CpG island density and genomic features such as number of chromosomes, chromosome size, and recombination rate. We also observed a trend of higher CpG island density in telomeric regions. Furthermore, we evaluated the performance of three computational algorithms for CpG island identifications. Finally, we compared our observations in mammals to other non-mammal vertebrates.

**Conclusion:**

Our study revealed that CpG islands vary greatly among mammalian genomes. Some factors such as recombination rate and chromosome size might have influenced the evolution of CpG islands in the course of mammalian evolution. Our results suggest a scenario in which an increase in chromosome number increases the rate of recombination, which in turn elevates GC content to help prevent loss of CpG islands and maintain their density. These findings should be useful for studying mammalian genomes, the role of CpG islands in gene function, and molecular evolution.

## Abstract 2

A systematic analysis of CpG islands in ten mammalian genomes suggests that an increase in chromosome number elevates GC content and prevents loss of CpG islands.

## Background

CpG islands (CGIs) are clusters of CpG dinucleotides in GC-rich regions and represent an important feature of mammalian genomes [1]. Mammalian genomic DNA generally shows a great deficit of CpG dinucleotides, for example, the ratio of the observed over the expected CpGs (Obs_CpG_/Exp_CpG_) is approximately 0.20-0.25 in the human and mouse genomes [2-4]. This deficit is largely attributed to the hypermutability of methylated CpGs to TpGs (or CpAs in the complementary strand) [5,6]. In comparison, CpGs in CGIs are often unmethylated and their frequencies are close to random expectation (for example, Obs_CpG_/Exp_CpG _= ~0.8 in the promoter-associated CGIs [7]). CGIs are often associated with the 5' end of genes and considered as gene markers [8,9]. However, a comparison of the human, mouse, and rat genomes indicated that, although these three genomes encode similar numbers of genes, the number of CGIs in the mouse (15,500) or rat (15,975) genome is far fewer than that (27,000) identified in the non-repetitive portions of the human genome [10-12]. The difference is probably due to a faster rate of loss of CGIs in the rodent lineage, rather than faster gains of CGIs in the human lineage [7,9]. However, it remains unclear whether the loss-of-CGI model holds for other mammalian genomes. Furthermore, to our best knowledge, there has been no comprehensive analysis of CGIs and their density at the DNA sequence level in mammals.

There are three major algorithms for identifying CGIs in a genomic sequence. The original algorithm was proposed by Gardiner-Garden and Frommer [13] in 1987; the three parameters are GC content >50%, Obs_CpG_/Exp_CpG _>0.60, and length >200 bp. This algorithm, often with some modifications, has been widely applied in the analysis of CGIs in single genes, small sets of genomic sequences, or single genomes. However, many repeats (for example, *Alu*), which are abundant in the vertebrate genome, also meet the criteria, so this algorithm has usually been used to scan CGIs only in non-repeat portions of the genome [2,11,12]. Second, Takai and Jones [14] evaluated the three parameters in Gardiner-Garden and Frommer's algorithm using human gene data and suggested an optimal set of parameters (GC content ≥55%, Obs_CpG_/Exp_CpG _≥0.65, and length ≥500 bp). This algorithm can effectively exclude false positive CGIs from repeats and more likely identify CGIs associate with the 5' end of human genes; it seems to be suitable for other genomes too [14]. Third, more recently, Hackenberg *et al*. [15] developed a new algorithm, namely CpGcluster, that entirely depends on the statistical significance of a CpG cluster from random sequences in the same chromosome. Because CpGcluster does not require a minimum length (for example, it identified CpG clusters as short as 8 bp) [15], it likely identifies many more CGIs (for example, 197,727 in the human genome) than other algorithms. In particular, CpGcluster may exaggerate the number of CGIs (that is, CpG clusters) in low GC-content chromosomes, which often have low gene density, because its CpG clusters were identified relative to the background (random) CpG property. Another similar CpG cluster algorithm identifies CpG clusters by requiring a minimum number of CpGs in each sequence fragment [16]. Since loss of CGIs is likely an evolutionary trend in at least some genomes [7,9,17], CpGcluster may be able to identify those CGIs that have undergone degradation and thus can not meet the criteria of Takai and Jones' or Gardiner-Garden and Frommer's algorithms.

Our major aim is to survey extant CGIs (that is, CGIs that meet the three typical criteria: length, GC content, and Obs_CpG_/Exp_CpG_) and their distribution in today's genomes, rather than to identify regions that might originally be CGIs, even though they do not meet the three typical criteria. A comparative study of the features of such CGIs will be helpful for studying the evolution of CGIs and sequence composition changes in the course of genome evolution. Recent genome sequencing projects have released a number of mammalian genomes with good quality annotations, but only few non-mammalian vertebrate genomes. Thus, in this study we focused on the analysis and comparison of CGIs and their correlations with genomic features in mammalian genomes. For our aim, it is appropriate to apply the same CGI detection algorithm to screen CGIs in multiple genomes for comparison. According to the introduction of the three algorithms above, we selected Takai and Jones' algorithm as a major algorithm in this study.

We conducted a systematic survey of CGIs in ten sequenced mammalian genomes: eight completely sequenced eutherian genomes (human (*Homo sapiens*), chimpanzee (*Pan troglodytes*), macaque (*Macaca mulatta*), mouse (*Mus musculus*), rat (*Rattus norvegicus*), dog (*Canis familiaris*), cow (*Bos taurus*), and horse (*Equus caballus*)); one completely sequenced metatherian genome (opossum (*Monodelphis domestica*)); and one prototherian genome (platypus (*Ornithorhynchus anatinus*)) whose sequence was completed with a 6× coverage, though it has not been completely assembled. We also compared the observations from these mammals to seven other non-mammal vertebrates.

## Results

### CGIs and CGI density in ten mammalian genomes

We first present our analysis of CGIs identified by Takai and Jones' algorithm [14] in ten mammalian genome sequences. The conclusions are essentially the same when we used the popular algorithm by Gardiner-Garden and Frommer [13] or the recently developed algorithm CpGcluster [15] (see Discussion). The species names and the sources of genome sequences are shown in the Materials and methods. Table 1 summarizes the genome information and statistics of CGIs. Except for the platypus, these genomes had similar sizes (2.0-3.3 Gb) and similar numbers of annotated genes (20,000-30,000; Additional data file 1). However, both the number of CGIs and the CGI density (measured by the average number of CGIs per Mb) vary greatly among genomes. The dog genome has the largest number of CGIs (58,327) and the platypus genome has the highest CGI density (35.9 CGIs/Mb). Remarkably, the number of CGIs in the dog genome is nearly three times that in the rat (19,568) or mouse (20,458) genome, even though the number of dog genes has been estimated to be smaller than those of human or mouse genes (dog, 19,300 [18]; human, 20,000-25,000 [19]; mouse, approximately 30,000 [11]). The CGI density (per Mb) ranges from 7.5 (opossum) to 35.9 (platypus) in the 10 genomes investigated. These results suggest that, although genes are often associated with CGIs, the extant CGIs are distributed very differently among genomic regions (for example, genes versus non-coding regions) in mammalian genomes.

**Table 1:** CpG islands and other genomic features in ten mammalian genomes

	Genome					CpG islands				
		
Species	Size (Gb)*	Number of chromosome pairs	Number of arms^†^	GC content (%)	Obs_CpG_/Exp_CpG_	Number of CGIs	CGI density (/Mb)	Avgerage length (bp)	GC content (%)	Obs_CpG_/Exp_CpG_
Human	2.85	23	82	40.9	0.236	37,531	13.2	1,089	62.0	0.743
Chimpanzee	2.75	24	84	40.7	0.233	35,845	13.0	1,011	60.3	0.761
Macaque	2.65	21	84	40.7	0.245	39,498	14.9	957	60.8	0.749
Mouse	2.48	20	40	41.7	0.192	20,458	8.2	1,043	60.6	0.756
Rat	2.48	21	64	41.9	0.220	19,568	7.9	1,004	59.7	0.758
Dog	2.31	39	80	41.0	0.244	58,327	25.3	1,102	62.2	0.753
Cow	2.29	30	62	41.9	0.236	36,729	16.0	1,023	61.2	0.740
Horse	2.03	32	92	41.0	0.285	33,135	16.3	937	59.2	0.749
Opossum	3.34	9	24	37.6	0.129	24,938	7.5	919	60.8	0.698
Platypus^‡^	0.41	26	NA	43.3	0.296	14,686	35.9	929	56.8	0.785

*The nucleotides marked as 'N' were not included in the analysis. ^†^Number of arms in a female. ^‡^Incomplete genome sequences (only 19 partially assembled chromosomes). NA, not available.

### Correlations between CGI density and other genomic features

**Figure 1:**
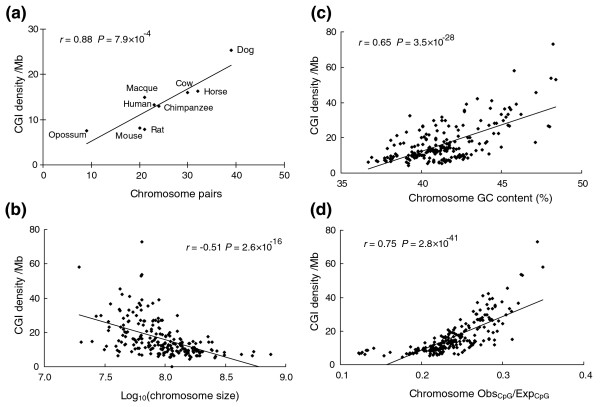
Correlations between CGI density and genomic features in nine mammalian genomes. The platypus chromosomes were excluded because of incomplete genome sequence data and chromosome data. **(a) **CGI density (per Mb) versus number of chromosome pairs. **(b) **CGI density (per Mb) versus log_10_(chromosome size). The Y chromosomes were excluded because of insufficient data. **(c) **CGI density (per Mb) versus chromosome GC content (%). **(d)** CGI density (per Mb) versus chromosome Obs_CpG_/Exp_CpG_.

We examined the correlations between CGI density and other genomic features. Because of incomplete genome sequence and lack of some chromosome data in platypus, we present the correlation results only for the other nine genomes; the conclusion will likely be the same when the platypus data become available (Additional data file 2). We found a highly significant positive correlation between CGI density and number of chromosome pairs in a genome (*r *= 0.88, *P *= 7.9 × 10^-4^; Figure 1a) and a significant correlation between CGI density and number of chromosome arms (*r *= 0.62, *P *= 0.037). As expected, there was a significant positive correlation between CGI density and Obs_CpG_/Exp_CpG _(*r *= 0.63, *P *= 0.035). No significant correlation was found between CGI density and genome size (*r *= -0.53, *P *= 0.073) or genome GC content (*r *= 0.24, *P *= 0.27).

There were a total of 219 chromosomes available in these 9 genomes after excluding the Y chromosomes. We found a highly significant negative correlation between CGI density and log_10_(chromosome size) (*r *= -0.51, *P *= 2.6 × 10^-16^; Figure 1b), a highly significant positive correlation between CGI density and GC content of the chromosome (*r *= 0.65, *P *= 3.5 × 10^-28^; Figure 1c), and a highly significant positive correlation between CGI density and Obs_CpG_/Exp_CpG _(*r *= 0.75, *P *= 2.8 × 10^-41^; Figure 1d). We further separated the chromosomes into different groups by their sizes (<25, 25-50, 50-75, 75-100, 100-150, 150-200, and >200 Mb). Interestingly, as the average size of a chromosome group increases, the CGI density decreases (Table 2). Indeed, the CGI density in small mammalian chromosomes (size <25 Mb) is, on average, about three times that in large chromosomes (size >200 Mb). We noted that the platypus (2n = 52), which has six pairs of large chromosomes but many small chromosomes [20], has a much higher CGI density than the other nine mammalian genomes (Table 1). These results are consistent with the previous observation that CGIs are highly concentrated on the microchromosomes in chickens [21].

**Table 2:** CGI densities in chromosomes with different sizes in nine mammalian genomes

Chromosome size (Mb)	Number of chromosomes	CGI density/Mb ± SD
<25	5	29.7 ± 17.7
25-50	35	24.0 ± 13.2
50-75	47	21.7 ± 11.3
75-100	43	14.7 ± 7.4
100-150	49	11.7 ± 4.6
150-200	26	9.7 ± 2.6
>200	14	9.4 ± 3.6
Total	219	16.4 ± 10.5

SD, standard deviation.

The dog has overall smaller chromosomes and high CGI density, while the opossum has a few large chromosomes and low CGI density. To check whether our correlation analysis was largely driven by these two species, we performed a similar analysis but excluded the dog and opossum data. The same conclusion still held. For example, we found a significant correlation between CGI density and number of chromosome pairs (*r *= 0.75, *P *= 0.026) and a significant correlation between CGI density and log_10_(chromosome size) (*r *= -0.49, *P *= 5.9 × 10^-12^).

CGIs are considered gene markers, so they are expected to highly correlate with gene density [2,22]. It is interesting to investigate whether the above correlation results still hold when gene information is excluded. We identified CGIs in the intergenic regions of nine mammalian genomes and found significant correlations between intergenic CGI density and log_10_(chromosome size) (*r *= -0.55, *P *= 7.3 × 10^-19^), GC content of the chromosome (*r *= 0.39, *P *= 8.6 × 10^-10^), and Obs_CpG_/Exp_CpG _(*r *= 0.67, *P *= 3.7 × 10^-30^). Details are shown in Additional data file 3.

It is also interesting to examine whether the correlations between CGI density and other genomic factors would hold in different genomic regions. We used human data because of their high quality annotations. According to gene annotations in the NCBI database, we identified 24,228 CGIs overlapped or within genes (gene-associated CGIs), 13,026 CGIs whose whole sequences were within intergenic regions (intergenic CGIs), 12,136 CGIs whose whole sequences were within gene regions (intragenic CGIs), and 11,192 CGIs overlapped with transcriptional start sites (TSS CGIs) in the human genome. Table 3 shows significant correlations between CGI density and genomic features (log_10_(chromosome size), GC content, and Obs_CpG_/Exp_CpG_) in all genomic regions when we compare the data at the chromosome level.

**Table 3:** Correlation between CGI density and genomic features in different human genomic regions

	Gene-associated CGIs (24,228)		Intergenic CGIs (13,026)		Intragenic CGIs (12,136)		TSS CGIs (11,192)	
				
	*r*	*P*	*r*	*P*	*r*	*P*	*r*	*P*
Log_10_(chromosome size)	-0.54	3.9 × 10^-3^	-0.55	3.4 × 10^-3^	-0.55	3.1 × 10^-3^	-0.51	7.0 × 10^-3^
GC content	0.88	1.7 × 10^-8^	0.87	2.9 × 10^-8^	0.85	1.9 × 10^-7^	0.91	5.4 × 10^-10^
Obs_CpG_/Exp_CpG_	0.92	1.5 × 10^-10^	0.91	8.3 × 10^-10^	0.92	2.5 × 10^-10^	0.91	1.0 × 10^-9^

Table 4 summarizes the correlations between CGIs and genomic features based on nine or ten genomes using three CGI identification algorithms.

**Table 4:** Summary of correlations between CGI density and genomic features

Algorithm	Genomic features	*r*	*P*	Shown in figure
TJ (9 genomes)	Chromosome pairs	0.88	7.9 × 10^-4^	1a
	Log_10_(chromosome size)	-0.51	2.6 × 10^-16^	1b
	Chromosome GC content	0.65	3.5 × 10^-28^	1c
	Chromosome Obs_CpG_/Exp_CpG_	0.75	2.8 × 10^-41^	1d
	Chromosome arms	0.62	0.037	
	Genome size	-0.53	0.073*	
	Genomic GC content	0.24	0.27*	
	Genomic Obs_CpG_/Exp_CpG_	0.63	0.035	
				
TJ (9 genomes, intergenic CGIs)	Chromosome pairs	0.79	0.005	S2a
	Log_10_(chromosome size)	-0.55	7.3 × 10^-19^	S2b
	Chromosome GC content	0.39	8.6 × 10^-10^	S2c
	Chromosome Obs_CpG_/Exp_CpG_	0.67	3.7 × 10^-30^	S2d
				
TJ (10 genomes)	Chromosome pairs	0.58	0.039	S1a
	Log_10_(chromosome size)	-0.70	2.6 × 10^-37^	S1b
	Chromosome GC content	0.64	3.7 × 10^-29^	S1c
	Chromosome Obs_CpG_/Exp_CpG_	0.89	1.5 × 10^-81^	S1d
				
GF (9 genomes)	Chromosome pairs	0.92	2.0 × 10^-4^	S5a
	Log_10_(chromosome size)	-0.63	1.3 × 10^-25^	S5b
	Chromosome GC content	0.72	3.2 × 10^-37^	S5c
	Chromosome Obs_CpG_/Exp_CpG_	0.81	2.4 × 10^-53^	S5d
				
CpGcluster (9 genomes)	Chromosome pairs	0.81	0.004	S6a
	Log_10_(chromosome size)	-0.52	1.6 × 10^-16^	S6b
	Chromosome GC content	0.21	0.001	S6c
	Chromosome Obs_CpG_/Exp_CpG_	0.61	5.5 × 10^-24^	S6d

*Insignificant correlation. GF, Gardiner-Garden and Frommer's algorithm; TJ, Takai and Jones' algorithm.

### CGI density and recombination rate

Recombination rate correlates with both the number of chromosomes and the number of chromosome arms, and elevates the GC content, probably via biased gene conversion [23,24]. Fine-scale recombination rates vary extensively among populations [25,26], genomic regions [27], or the homologous regions between two closely related organisms (human and chimpanzee) [28,29], suggesting a rapid evolution of local pattern of recombination rates. Many genomic features, including CpG dinucleotide frequencies (but not CGIs or CGI density) in genomic sequences, have been employed to analyze the pattern of recombination rate. Here we examined specifically the relationship between CGI density and recombination rate at the genome level. We retrieved human recombination rate data (window size, 1 Mb, 2,772 windows) from the UCSC Genome Browser [30]. We found a significant positive correlation between CGI density and recombination rate (*r *= 0.18, *P *= 1.1 × 10^-22^).

**Figure 2:**
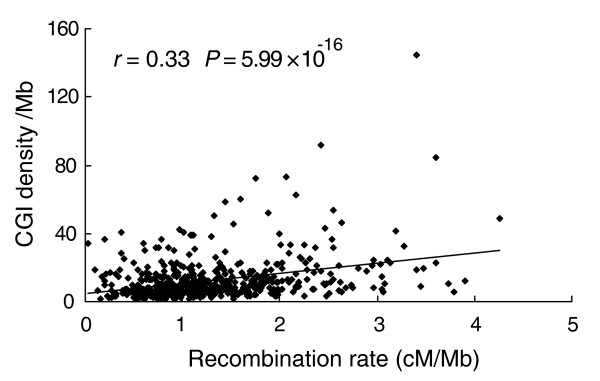
Correlation between CGI density and recombination rate (cM/Mb) in the human genome; a 5 Mb window was used.

We obtained another set of recombination rate data (in 5 Mb and 10 Mb windows) for the human, mouse and rat from Jensen-Seaman *et al*. [31]. We discarded those regions that had more than 50% 'N's ('N' denotes an uncertain nucleotide in the sequence) or whose recombination rate was 0. In the latter case, it was likely due to insufficient available genetic markers or a small number of meioses used to construct the genetic maps [31]. Again, we found a significant correlation between CGI density and recombination rate, regardless of window size (5 Mb or 10 Mb; Table 5 and Additional data file 4). For example, the correlation coefficient was 0.33 (*P *= 5.9 × 10^-16^) for human recombination rates measured in a 5 Mb window (Figure 2). The correlation became stronger as the window size increased. Furthermore, the extent of the correlation was different among the three genomes. For example, the coefficients were 0.33 (human), 0.24 (mouse), and 0.17 (rat), respectively, when the 5 Mb window was used.

**Table 5:** Correlation between CGI density and recombination rate in human, mouse and rat

	Window size (Mb)	*r*	*P*
Human	1	0.18	1.1 × 10^-22^
	5	0.33	5.9 × 10^-16^
	10	0.40	1.7 × 10^-12^
			
Mouse	5	0.24	3.6 × 10^-7^
	10	0.33	8.0 × 10^-8^
			
Rat	5	0.17	8.1 × 10^-5^
	10	0.26	1.7 × 10^-5^

The detailed distributions are shown in Additional data file 4. Human recombination rate data measured with a 1 Mb window were based on the deCODE genetic map and downloaded from the UCSC Genome Browser [30]. Recombination rate data measured with 5 Mb and 10 Mb windows were prepared by Jensen-Seaman *et al*. [31] and downloaded from the associated supplementary material website.

**Figure 3:**
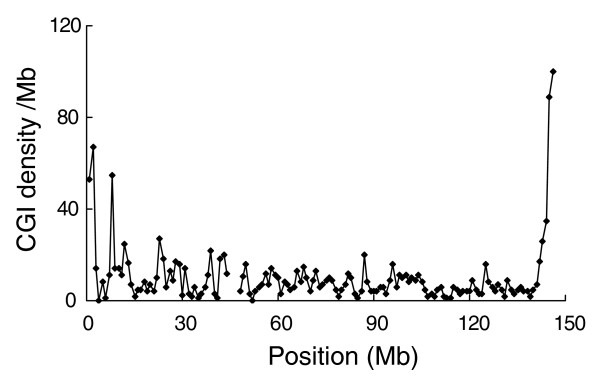
Distribution of CGI density (per Mb) on human chromosome 8. The data indicate a trend of higher CGI density in telomeric regions.

Recombination rates were found to increase from the centromeric towards telomeric regions [31]. Interestingly, we observed a trend of higher CGI density in the telomeric regions (Figure 3) in many chromosomes. This feature supports a positive correlation between CGI density and recombination rate. However, this finding is opposite to a previous observation of no correlation between CGI features and chromosomal telomere position based on a small gene dataset [17].

### Comparison of CGIs in non-mammalian vertebrate genomes

To retrieve information on the CGIs in vertebrate genomes, we scanned CGIs in seven non-mammalian vertebrate genomes, including the chicken, lizard and five fish (tetraodon, medaka, zebrafish, stickleback and fugu) genomes. Except for lizard and fugu, all these genomes had assembled chromosomes.

Table 6 shows the CGIs and other genome information for the seven non-mammalian vertebrates. The CGI density had a much wider range (14.7-161.6 per Mb) among these genomes. The CGI densities in the chicken (23.0 per Mb) and green anole lizard (25.9 per Mb) were similar to that in the dog (25.3 per Mb), higher than that in the other eight therians, but lower than that (35.9 per Mb) in the platypus (prototherian) (Table 1). It is worth noting that both the chicken and platypus have many small chromosomes. The chicken karyotype consists of 39 chromosomes, of which 33 are classified as microchromosomes [32]. At the DNA sequence level, chicken chromosomes were separated into three groups (large macrochromosomes, intermediate chromosomes and microchromosomes) by the International Chicken Genome Sequencing Consortium [33]. Using this classification, we found that CGI density in the 20 chicken microchromosomes (51.7 per Mb) was much higher than that (15.0 per Mb) in the 6 large macrochromosomes (Table 6), consistent with an earlier report [21]. We did not estimate the CGI density in the large or small chromosomes of platypus because the available assembled genome sequences (410 Mb) represent only a small portion of the genome, which is expected to be about the same size as the human genome [20].

**Table 6:** CpG islands and other genomic features in non-mammalian genomes

	Genome				CpG islands				
		
Species	Length (Mb)*	Number of chromosome pairs	GC content (%)	Obs_CpG_/Exp_CpG_	Number of CGIs	CGI density (/Mb)	Avgerage length (bp)	GC content (%)	Obs_CpG_/Exp_CpG_
Chicken^†^	985	39	41.4	0.248	22,623	23.0	1,098	60.0	0.844
Microchromosome	167	20	45.7	0.305	8,634	51.7	1,040	60.4	0.810
Macrochromosome	674	6	40.0	0.219	10,125	15.0	1,138	59.6	0.863
Lizard	1,742	18	40.4	0.296	45,171	25.9	899	56.8	0.728
Tetraodon	187	21	45.9	0.601	30,175	161.6	1,013	56.7	0.782
Stickleback	391	21	44.5	0.662	61,768	157.8	824	55.8	0.842
Medaka	582	24	40.1	0.479	21,522	37.0	746	55.8	0.784
Zebrafish	1,524	25	36.5	0.531	22,392	14.7	1,162	57.0	0.869
Fugu	351	22	45.5	0.565	47,251	134.5	872	56.0	0.808

*The nucleotides marked as 'N' were not included in the analysis. ^†^Only 30 chromosomes were used in the analysis because chromosomes 29-31 and 33-38 were too small to assemble [39]. The microchromosomes included chromosomes GGA11-28, 32 and W and the macrochromosomes included chromosomes GGA1-5 and Z.

CGI densities in the five fish genomes varied to a much greater extent than in the mammalian genomes. The CGI densities in tetraodon (161.6 per Mb) and stickleback (157.8 per Mb) were about 11 times that in zebrafish (14.7 per Mb). The Obs_CpG_/Exp_CpG _ratios in the fish genomes (0.479-0.662) were also much higher than those (0.129-0.296) in the mammalian, the chicken (0.248) and the lizard (0.296) genomes. Fishes are cold-blooded vertebrates and lack GC-rich isochores [34]. An early study found certain fish did not have elevated GC content in nonmethylated CGIs [35], so our comparison of CGIs in fishes should be taken with caution.

**Figure 4:**
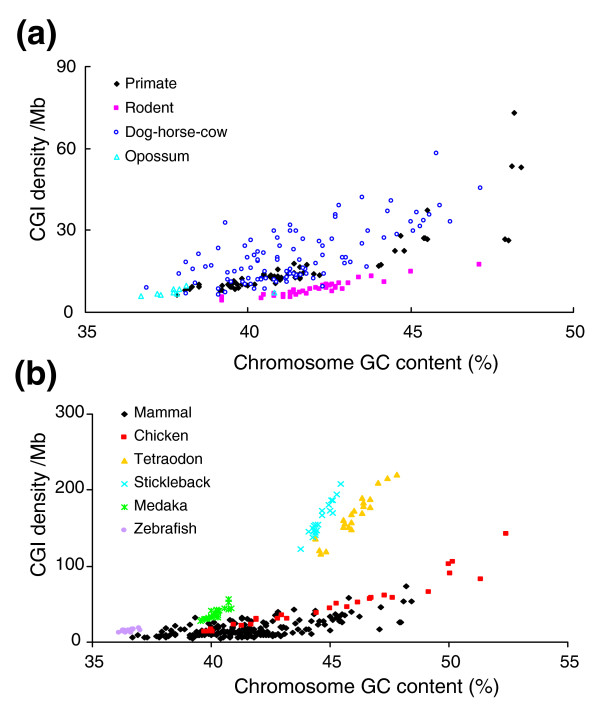
CGI density comparison between mammals and non-mammals. This figure shows the distribution of CGI density (per Mb) versus chromosome GC content (%). **(a) **Comparison of four groups in mammals. **(b) **Comparison of mammals, chicken and fish.

In contrast to the observation in mammalian genomes, the correlation between CGI density and number of chromosome pairs in the seven non-mammals was not significant (*r *= -0.42, *P *= 0.17). We further examined CGI density at the chromosome level in the five non-mammalian genomes (chicken, tetraodon, stickleback, medaka and zebrafish), whose assembled chromosomes are available, and compared it to the nine mammalian genomes. To distinguish the features of CGIs among different genomes, we separated them into different groups: primates (human, chimpanzee and macaque), rodents (mouse and rat), dog-horse-cow, opossum, chicken and fish (tetraodon, stickleback, medaka and zebrafish). Figure 4 shows the plots of CGI density over chromosome GC content. Although there is an overall trend of increasing CGI density with chromosome GC content in both the mammals and non-mammals, their distributions of CGI densities over the chromosome GC content are different. In mammals, CGI density is high in dog-horse-cow and low in rodents, but extensive overlaps are seen among different groups, especially between primates and other groups (Figure 4a). This pattern is more evident in the plots of CGI density versus log_10_(chromosome size) or versus chromosome Obs_CpG_/Exp_CpG _ratios (Additional data file 5). Interestingly, we found an overall distinct distribution pattern among non-mammal genomes, especially among the fish genomes (Figure 4b). The chromosomes from each fish genome clustered but they were separated from other fish genomes (Figure 4b, Additional data file 5). Finally, when all species were plotted together, there were overlaps between mammals and non-mammals, but overall, fish chromosomes and chicken microchromosomes could be separated from the mammalian chromosomes (Figure 4b, Additional data file 5).

## Discussion

### Influence of CGI identification algorithms

There are three major algorithms for identifying CGIs in a genomic sequence (reviewed in the Background). The major aim in this study is to investigate and compare the CGIs in today's mammalian genomes, rather than to identify CGIs in the mammalian ancestral sequences. Thus, our analysis may provide insights into how CGIs have evolved and their association with gene function and other genomic factors. Since CGIs have been widely documented to be approximately 1 kb long [2,6], Takai and Jones' stringent criteria seem to be the most appropriate for our analysis. To assure the reliability of our analysis, we performed similar analysis using Gardiner-Garden and Frommer's algorithm (only on the non-repeat portions of the genomes) and CpGcluster with the ten mammalian genomes and seven other vertebrate genomes under study. The conclusions were the same; see detailed results in Table 4 and Additional data files 6 and 7. For example, there was a significant positive correlation between CGI density and chromosome number, using Gardiner-Garden and Frommer's algorithm (*r *= 0.92, *P *= 2.0 × 10^-4^; Additional data file 6) or CpGcluster (*r *= 0.81, *P *= 0.004; Additional data file 7).

However, we found that the number of CGIs identified by CpGcluster or Gardiner-Garden and Frommer's algorithm was remarkably larger than that identified by Takai and Jones' algorithm (Additional data file 8); for example, the numbers of CGIs identified in the human genome was 37,531 (Takai and Jones), 76,678 (Gardiner-Garden and Frommer), and 197,727 (CpGcluster). The number of genes was estimated to be approximately in the range 20,000-30,000 in mammalian genomes (Additional data file 1). Since CGIs have been widely considered as gene markers, both the Gardiner-Garden and Frommer algorithm and CpGcluster likely identified either many CGIs that are not associated with genes or multiple CGIs that share one gene. To address the latter case, we evaluated the length distribution of CGIs identified by the three algorithms. Among all these vertebrate genomes, the majority of CGIs identified by CpGcluster were shorter than 500 bp (Additional data file 8), which is the minimum length in Takai and Jones' algorithm. For example, the proportions of human CGIs identified by CpGcluster were 44.3% (<200 bp), 45.9% (200-500 bp), 7.3% (500-1,000 bp), 1.9% (1,000-1,500 bp), 0.4% (1,500-2,000 bp), and 0.2% (≥2,000 bp). For Gardiner-Garden and Frommer's algorithm, the proportion of CGIs shorter than 500 bp was also large, for example, 65.8% in the human CGIs and 64.8% in the opossum CGIs (Additional data file 8). Based on the evaluation above, we consider that our analysis using Takai and Jones' algorithm is the most reliable and appropriate, though further evaluation of species-specific algorithms may enhance our results.

### Evolution of CGIs

It was hypothesized that CGIs arose once at the dawn of vertebrate evolution and vertebrate ancestral genes were embedded in entirely non-methylated DNA during the divergence of vertebrates [9]. Genome-wide methylation has been found to be common in vertebrates (except for promoter-associated CGIs) and fractional methylation common in invertebrates. The transition from fractional to global methylation likely occurred around the origin of vertebrates [36]. Many CGIs might have lost their typical features due to *de novo *methylation at their CpG sites and subsequent high deamination rates at the newly methylated CpG sites, leading to TpG and CpA dinucleotides. Excess of TpGs and CpAs as well as other vanishing CGI features (decreasing length, Obs_CpG_/Exp_CpG _ratio and GC content) has been found in the homologous gene regions, evidence of frequent CGI losses in mouse and human genes and a faster loss rate in mice [7,9,17]. Recent methylation studies revealed weak CGIs in promoter regions (promoters with intermediate CpG content, ICPs), most of which were not found in the CGI library, had a faster loss rate of CpGs than stronger CGIs (promoters with high CpG content, HCPs), suggesting that strong CGIs might be protected from methylation and are thus better conserved during evolution [22,37,38]. Using the data in Weber *et al*. [37] and Mikkelsen *et al*. [38], we found that HCP density has stronger correlations with genomic features than ICPs in both the human and mouse genomes. The CGIs identified by the Takai-Jones algorithm are different from HCPs or ICPs. However, when we separated the promoter-associated CGIs identified by the Takai-Jones algorithm into HCGIs (those that satisfied the HCP criteria) and non-HCGIs, we also found that HCGIs had stronger correlations with genomic features than non-HCGIs. This supports the observations from the methylation studies mentioned above. Although loss of CGIs is likely a major evolutionary scenario in mammals, little comparative analysis at the DNA sequence level has been performed yet, because CGIs have been thought to be poorly conserved between species [7,9]. Our CGI analysis indicated that rodents have the lowest CGI density and most other eutherians have moderate CGI density when compared to platypus (Table 1). Platypus is one of the only three extant monotremes and has a fascinating mixture of features typical of mammals and of reptiles and birds. Monotremes (mammalian subclass Prototheria) are the oldest branch of the mammalian tree, diverging 210 million years ago from the therian mammals [20]. Although the platypus genome is incomplete, its higher CGI density is likely true because high frequencies of GC and CG dinucleotides and high GC content have been reported [20]. Further, our analysis of the chicken (bird) and green anole lizard genomic sequences, the only reptilian genome available at present, showed higher CGI density than most of the therians (except dogs) we examined. These data support an overall decrease in CGIs in mammalian genomes.

Below we discuss specific CGI features of a few species. The low number of CGIs in the rodent genome is likely due to a much higher rate of CGI loss and a weaker selective constraint in the rodent lineage [7,17]. Interestingly, the dog has a notably large number of CGIs and high CGI density among the nine therians investigated. Our further analysis revealed that the difference is due to the substantial enrichment of CGIs in dog's intergenic and intronic regions, while the number of CGIs associated with the 5' end of genes is similar to the human and the mouse (data not shown). Whether and how CGIs have accumulated in dog requires further investigation. It is also worth noting that opossum, which belongs to metatheria, is another evolutionarily ancient lineage of mammals. The CGI density is very low (7.5 per Mb). This is likely attributed to its large chromosomes (Table 1), as large chromosomes are correlated with low CGI density (Figure 1). Large chromosomes reduce recombination rate, which has a positive correlation with CGI density (Figure 2).

### Other possible factors that might influence CGI density

**Figure 5:**
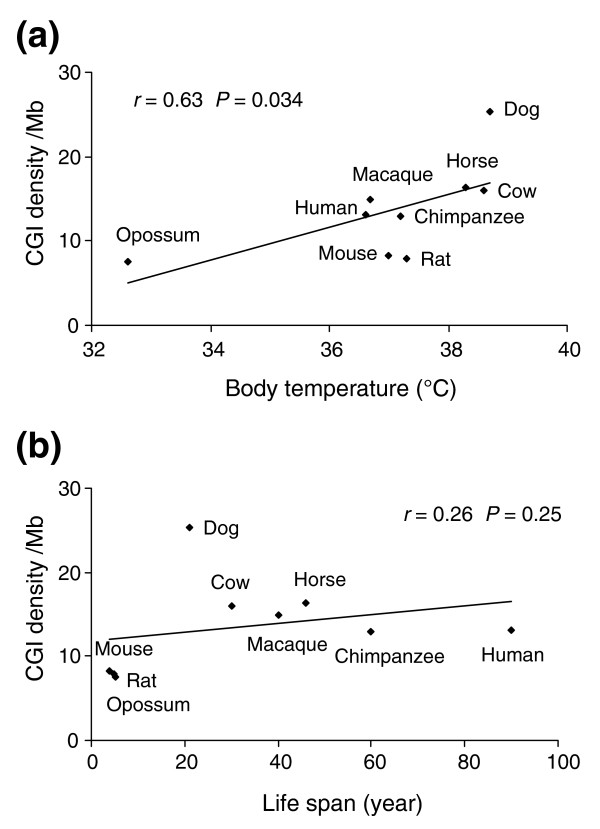
Correlation between CGI density and other genetic factors. **(a) **Significant correlation between CGI density and body temperature. **(b) **Insignificant correlation between CGI density and lifespan.

It is interesting to examine whether species traits such as lifespan, body temperature and body mass are related to CGI density. The small body size and short lifespan of mice were speculated to allow for their tolerance towards leaky control of gene activity, including erosion of CGIs [17]. A previous study also revealed that methylation status is correlated with body temperatures in fish and affected by the local environment [39]. It was also proposed that GC content of the isochores is driven by increasing body temperature, which has selective advantages because of being more thermally stable in higher GC-content regions [40]. Our correlation analysis found a significant correlation between CGI density and body temperature in eight eutherians (*r *= 0.67, *P *= 0.035) and nine therians (*r *= 0.63, *P *= 0.034; Figure 5a). However, when platypus and/or chicken were added, the correlation became insignificant. Furthermore, we did not find a significant correlation between CGI density and lifespan in the eight eutherians (*r *= 0.14, *P *= 0.38) or nine therians (*r *= 0.26, *P *= 0.25; Figure 5b). Some factors might have affected the estimation of lifespan, making the analysis unreliable. First, living environments are much different between domesticated and wild animals; meanwhile, modern medical treatment has increased human longevity. Second, lifespan in the same species may differ according to factors such as sex [41] and hormonal regulation [42,43]. Third, the divergence among mammals is low when compared to other vertebrates. In summary, our analysis of these species traits should be considered preliminary.

## Conclusion

This study represents a systematic comparative genomic analysis of CGIs and CGI density at the DNA sequence level in mammals. It reveals significant correlations between CGI density and genomic features such as number of chromosome pairs, chromosome size, and recombination rate. Our results suggest a genome evolution scenario in which an increase in chromosome number increases the rate of recombination, which in turn elevates GC content to help prevent loss of CGIs and maintain CGI density. We compared CGI features in other non-mammalian vertebrates and discussed other factors such as body temperature and lifespan that have previously been speculated to influence sequence composition evolution.

## Materials and methods

### Genome sequences and genome information

We downloaded the assembled genome sequences (ten mammalian genomes and seven non-mammalian vertebrate genomes) from the National Center for Biotechnology Information (NCBI) [44] and the UCSC Genome Browser [30]. The species names and data sources are provided in Table 7. The repeat-masked sequences of these genomes were downloaded from the UCSC Genome Browser [30]. We used the EMBOSS package [45] to calculate the genome size, the GC content and the Obs_CpG_/Exp_CpG _ratios. Gene numbers were based on the annotations in Ensembl [46] and also in the literature (details are shown in Additional data file 1). At present, it remains a great challenge to obtain an accurate estimation of the gene number in a genome, but we suspect that the actual gene numbers in these genomes are likely in a smaller range than the range 20,000-30,000 in Additional data file 1.

**Table 7:** Names and sequence information of ten mammals and other vertebrates

Common name	Species name	Sequence build	Data source
Mammal			
Human	*Homo sapiens*	35.1	NCBI [44]
Chimpanzee	*Pan troglodytes*	2.1	NCBI [44]
Macaque	*Macaca mulatta*	1.1	NCBI [44]
Mouse	*Mus musculus*	34.1	NCBI [44]
Rat	*Rattus norvegicus*	4.1	NCBI [44]
Dog	*Canis familiaris*	2.1	NCBI [44]
Cow	*Bos taurus*	3.1	NCBI [44]
Horse	*Equus caballus*	1.1	NCBI [44]
Opossum	*Monodelphis domestica*	2.1	NCBI [44]
			
Platypus*	*Ornithorhynchus anatinus*	1.1	NCBI [44]
			
Non-mammal vertebrate			
Chicken^†^	*Gallus gallus*	2.1	NCBI [44]
Green anole lizard^‡^	*Anolis carolinensis*	anoCar1	UCSC [30]
Tetraodon	*Tetraodon nigroviridis*	tetNig1	UCSC [30]
Stickleback	*Gasterosteus aculeatus*	gasAcu1	UCSC [30]
Medaka	*Oryzias latipes*	oryLat1	UCSC [30]
Zebrafish	*Danio rerio*	danRer5	UCSC [30]
			
Fugu^‡^	*Takifugu rubripes*	fr2	UCSC [30]

*The platypus genome was partially assembled. Only chromosomes 1-7, 10-12, 14, 15, 17, 18, 20, X1-X3, and X5 were available. ^†^Only chromosomes 1-28, 32, W, and Z were available. ^‡^No assembled chromosomes.

### Identification of CpG islands

We used three algorithms to identify CGIs. First, we used the stringent search criteria in the Takai and Jones algorithm [14]: GC content ≥55%, Obs_CpG_/Exp_CpG _≥0.65, and length ≥500 bp. Second, we used the algorithm originally developed by Gardiner-Garden and Frommer [13]: GC content >50%, Obs_CpG_/Exp_CpG _>0.60, and length >200 bp. Because some repeats (for example, *Alu*) meet these criteria, we scanned CGIs in the non-repeat portions of these genomes only, as similarly done in other genome-wide identification studies [2,11]. For both the Takai and Jones and the Gardiner-Garden and Frommer algorithms, we used the CpG island searcher program (CpGi130) available at [47]. Third, we used CpGcluster developed by Hackenberg *et al. *[15] to scan CGIs in the whole genome.

We used the method of Jiang and Zhao [48] to identify CGIs in different genomic regions (genes, intergenic regions, intragenic regions, and TSS regions). Briefly, we compared the locations of CGIs with the coordinates of genic, intergenic, and intragenic regions and TSSs based on the human gene annotation information from the NCBI database (build 35.1) [44,49]. CGIs overlapped with any genes were classified as gene-associated CGIs; CGIs whose whole sequences were in intergenic regions were classified as intergenic CGIs; CGIs whose sequences were in gene regions were classified as intragenic CGIs; and CGIs overlapped with TSSs were classified as TSS CGIs.

### Recombination rate and CGI density

We retrieved human recombination rate data based on the deCODE genetic map [50] from the UCSC Genome Browser [30]. The recombination rates were measured in 1 Mb windows. We obtained another set of recombination rates from Jensen-Seaman *et al*. [31]. These data were measured in 5 Mb and 10 Mb windows for the human, mouse and rat and are available in the supplementary material for Jensen-Seaman *et al*. [31]. For both datasets, we discarded those regions having more than 50% 'N's [31]. We also discarded those regions whose recombination rates were 0 because of too few genetic markers found in these regions [31].

### Body temperature and lifespan in mammals

Records of body temperature in a species may vary to some extent in the literature because they might be measured in different environments (for example, time of day, season, or geographical location) or different sites of the body. The body temperatures of ten mammals in this study were obtained from the literature (details are shown in Additional data file 9). When a species has a range of body temperatures in the literature, the average was used as the representative temperature. There are several measurements of lifespan, such as maximum lifespan, average lifespan, and lifespan of each sex. We used maximum lifespan, which was based on reports in the literature and from the AnAge database [51] (Additional data file 9).

## Abbreviations

CGI, CpG island; HCGI, CGI satisfying the HCP criteria; HCP, high CpG content promoter; ICP, intermediate CpG content promoter; TSS, transcriptional start site.

## Authors' contributions

LH prepared the data, carried out the data analysis, and contributed to the writing of the manuscript. BS participated in study design and coordination. WHL participated in study design and contributed to the writing of the manuscript. ZZ conceived of the study, participated in the data analysis and interpretation, and contributed to the writing of the manuscript. All authors read and approved the final manuscript.

## Additional data files

The following additional data are available. Additional data file 1 is a table that lists the numbers of genes estimated in mammalian genomes. Additional data file 2 shows the correlations between CGI density and genomic features in ten mammalian genomes (including platypus). Additional data file 3 shows the correlations between intergenic CGI density and genomic features in nine mammalian genomes. Additional data file 4 shows the correlations between CGI density and average recombination rate (cM/Mb) in the human, mouse and rat genomes. Additional data file 5 provides the comparison of CpG islands and other genomic features between mammalian and non-mammalian genomes. Additional data file 6 shows the correlations between CGI density and genomic features in mammalian genomes using the Gardiner-Garden and Frommer algorithm in the non-repeat portions of genomes. Additional data file 7 shows the correlations between CGI density and genomic features in mammalian genomes using the CpGcluster algorithm. Additional data file 8 lists the numbers of CGIs in each genome identified by the three algorithms and shows their length distribution. Additional data file 9 lists the body temperature and lifespan for each species.

## Supplementary Material

Additional file 1 Numbers of genes estimated in mammalian genomes. Click here for file

Additional file 2 Correlations between CGI density and genomic features in ten mammalian genomes (including platypus). Click here for file

Additional file 3 Correlations between intergenic CGI density and genomic features in nine mammalian genomes. Click here for file

Additional file 4 Correlations between CGI density and average recombination rate (cM/Mb) in the human, mouse and rat genomes. Click here for file

Additional file 5 Comparison of CpG islands and other genomic features between mammalian and non-mammalian genomes. Click here for file

Additional file 6 Correlations between CGI density and genomic features in mammalian genomes using the Gardiner-Garden and Frommer algorithm in the non-repeat portions of genomes. In both Additional data files 6 and 7, the platypus chromosomes were excluded because of incomplete genome sequence data and chromosome data. The conclusion would be the same when the platypus data were included. Click here for file

Additional file 7 Correlations between CGI density and genomic features in mammalian genomes using the CpGcluster algorithm. In both Additional data files 6 and 7, the platypus chromosomes were excluded because of incomplete genome sequence data and chromosome data. The conclusion would be the same when the platypus data were included. Click here for file

Additional file 8 The first sheet ('overview') summarizes the total number of CGIs in each genome identified by each algorithm. The length distribution of CGIs in each genome is shown in each additional sheet. Click here for file

Additional file 9 Body temperature and lifespan for each species. Click here for file
