# Supplementary material for: CpG island density and its correlations with genomic features in mammalian genomes
Source: Genome Biol. 2008 May 13;9(5):R79. doi: 10.1186/gb-2008-9-5-r79 (PMC2441465; doi:10.1186/gb-2008-9-5-r79)
Supplement: Additional file 1 — Numbers of genes estimated in mammalian genomes. [file gb-2008-9-5-r79-S1.pdf]

Gene numbers were based on the annotations in the Ensembl database (build 49) and also in literature. At present, it still remains a great challenge to obtain an accurate estimation of gene number in a genome, but we suspect that the actual gene numbers in these genomes are likely in a smaller range than the range 20,000 – 30,000 in table S1.

Table S1 Number of genes in mammalian genomes

Species	Ensembl						Estimates in literature
	Known genes	Projected genes	Novel genes	Psudogenes	RNA genes	**Total**	
Human	22,658	N/A	82	2,081	4,421	**29,242**	20,000 – 25,000 [1][a]<br>20,500 [2]<br>19,700 [3]
Chimpanzee	2,577	16,858	1,108	872	4,240	**25,655**	16,045 [4][b]
Macaque	874	17,340	3,691	1,762	4,003	**27,670**	~20,000 – 25,000 [5][c]
Mouse	22,010	N/A	1,483	1,190	3,014	**27,697**	~30,000 [6][d]
Rat	17,738	1,919	2,846	1,756	2,924	**27,183**	20,973 [7][e]
Dog	1,461	14,198	3,646	1,742	2,448	**23,495**	19,300 [8][f]
Cow	16,938	1,781	3,027	1,264	2,496	**25,506**	N/A
Horse	746	13,601	6,390	4,443	N/A	**25,180**	N/A
Opossum	119	13,935	5,417	722	1,251	**21,444**	18,000 – 20,000 [9][g]
Platypus	54	10,866	7,031	547	3,074	**21,572**	N/A

[a]Protein-coding genes.
[b]Based on the alignment to human RefSeq transcripts.
[c]Predicted by Ensembl gene models, Gnomen (NCBI) and Nscan.
[d]Protein-coding genes.
[e]Predicted by the Ensembl gene prediction pipeline.
[f]Based on evidence-based method.
[g]Protein-coding genes.

References

1. **Finishing the euchromatic sequence of the human genome.** *Nature* 2004, **431:**931-945.
2. Clamp M, Fry B, Kamal M, Xie X, Cuff J, Lin MF, Kellis M, Lindblad-Toh K, Lander ES: **Distinguishing protein-coding and noncoding genes in the human genome.** *Proc Natl Acad Sci USA* 2007, **104:**19428-19433.
3. Goodstadt L, Ponting CP: **Phylogenetic reconstruction of orthology, paralogy, and**

conserved synteny for dog and human. *PLoS Comput Biol* 2006, **2:**e133.
4. **Initial sequence of the chimpanzee genome and comparison with the human genome.** *Nature* 2005, **437:**69-87.
5. Gibbs RA, Rogers J, Katze MG, Bumgarner R, Weinstock GM, Mardis ER, Remington KA, Strausberg RL, Venter JC, Wilson RK, *et al.*: **Evolutionary and biomedical insights from the rhesus macaque genome.** *Science* 2007, **316:**222-234.
6. Waterston RH, Lindblad-Toh K, Birney E, Rogers J, Abril JF, Agarwal P, Agarwala R, Ainscough R, Alexandersson M, An P, *et al.*: **Initial sequencing and comparative analysis of the mouse genome.** *Nature* 2002, **420:**520-562.
7. Gibbs RA, Weinstock GM, Metzker ML, Muzny DM, Sodergren EJ, Scherer S, Scott G, Steffen D, Worley KC, Burch PE, *et al.*: **Genome sequence of the Brown Norway rat yields insights into mammalian evolution.** *Nature* 2004, **428:**493-521.
8. Lindblad-Toh K, Wade CM, Mikkelsen TS, Karlsson EK, Jaffe DB, Kamal M, Clamp M, Chang JL, Kulbokas III EJ, Zody MC, *et al.*: **Genome sequence, comparative analysis and haplotype structure of the domestic dog.** *Nature* 2005, **438:**803-819.
9. Mikkelsen TS, Wakefield MJ, Aken B, Amemiya CT, Chang JL, Duke S, Garber M, Gentles AJ, Goodstadt L, Heger A, *et al.*: **Genome of the marsupial Monodelphis domestica reveals innovation in non-coding sequences.** *Nature* 2007, **447:**167-177.
