# Supplementary material for: CpG island density and its correlations with genomic features in mammalian genomes
Source: Genome Biol. 2008 May 13;9(5):R79. doi: 10.1186/gb-2008-9-5-r79 (PMC2441465; doi:10.1186/gb-2008-9-5-r79)
Supplement: Additional file 2 — Correlations between CGI density and genomic features in ten mammalian genomes (including platypus). [file gb-2008-9-5-r79-S2.pdf]

Figure S1 Correlations between CGI density and genomic features in 10 mammalian genomes (**including platypus**). **(A)** CGI density (per Mb) vs. number of chromosome pairs. **(B)** CGI density (per Mb) vs. $\log_{10}$(chromosome size). **(C)** CGI density (per Mb) vs. chromosome GC content (%). **(D)** CGI density (per Mb) vs. chromosome Obs_{CpG}/Exp_{CpG}.

A

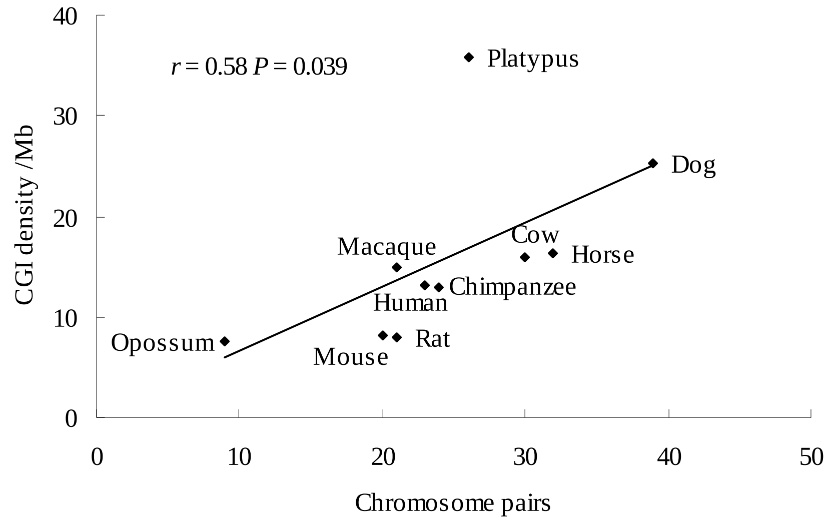

B

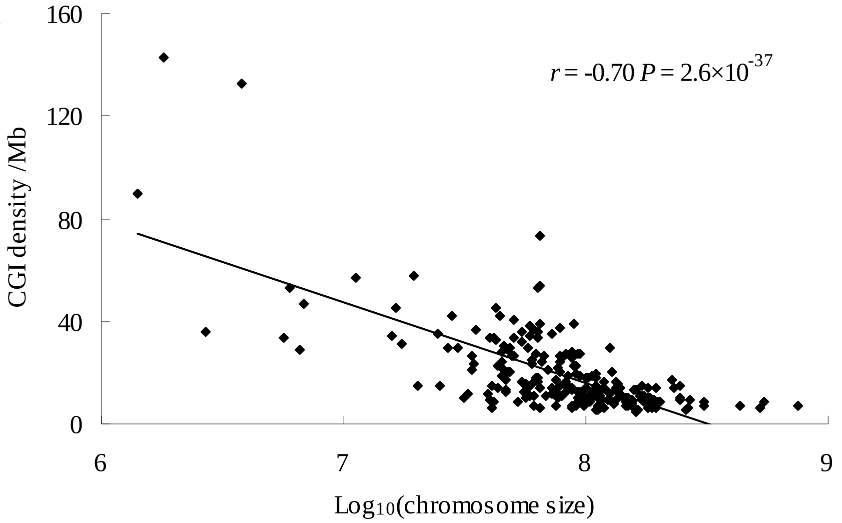

C

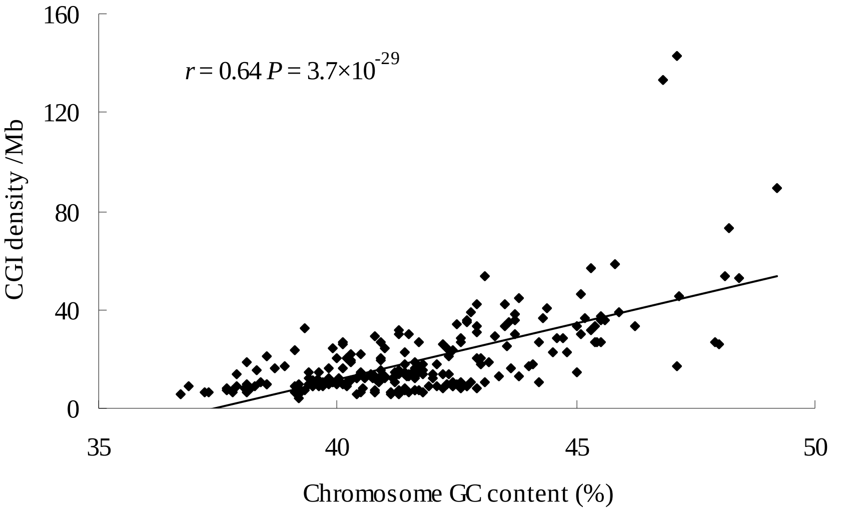
r = 0.64 P = 3.7×10-29
CGI density /Mb
0
40
80
120
160
35
40
45
50
Chromosome GC content (%)

D

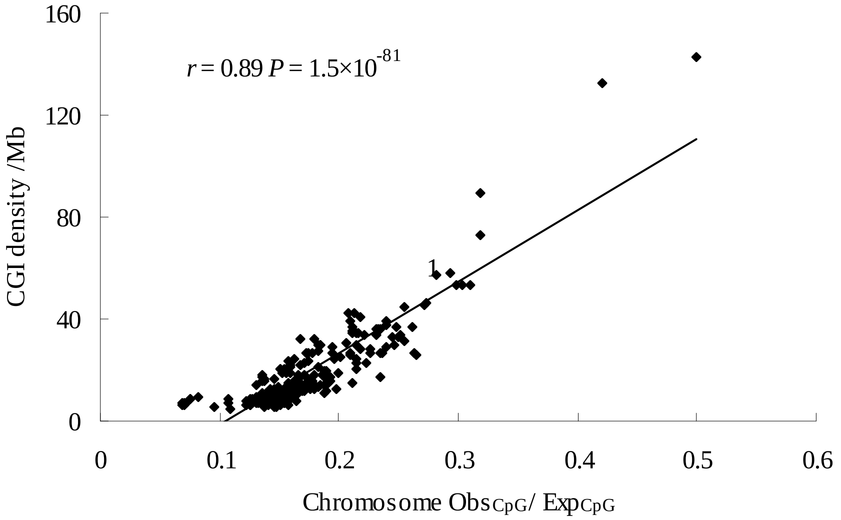
r = 0.89 P = 1.5×10-81
CGI density /Mb
0
40
80
120
160
1
0
0.1
0.2
0.3
0.4
0.5
0.6
Chromosome ObsCpG/ ExpCpG
