# Supplementary material for: CpG island density and its correlations with genomic features in mammalian genomes
Source: Genome Biol. 2008 May 13;9(5):R79. doi: 10.1186/gb-2008-9-5-r79 (PMC2441465; doi:10.1186/gb-2008-9-5-r79)
Supplement: Additional file 3 — Correlations between intergenic CGI density and genomic features in nine mammalian genomes. [file gb-2008-9-5-r79-S3.pdf]

Figure S2 Correlations between **Intergenic CGI** density and genomic features in 9 mammalian genomes **(A)** Intergenic CGI density (per Mb) vs. number of chromosome pairs. **(B)** Intergenic CGI density (per Mb) vs. $\log_{10}$(chromosome size). **(C)** Intergenic CGI density (per Mb) vs. chromosome GC content (%). **(D)** Intergenic CGI density (per Mb) vs. chromosome Obs_{CpG}/Exp_{CpG}.

A

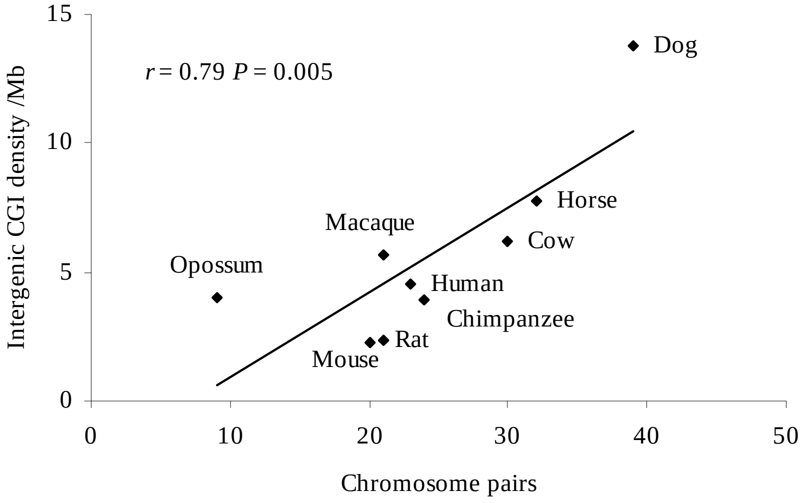

B

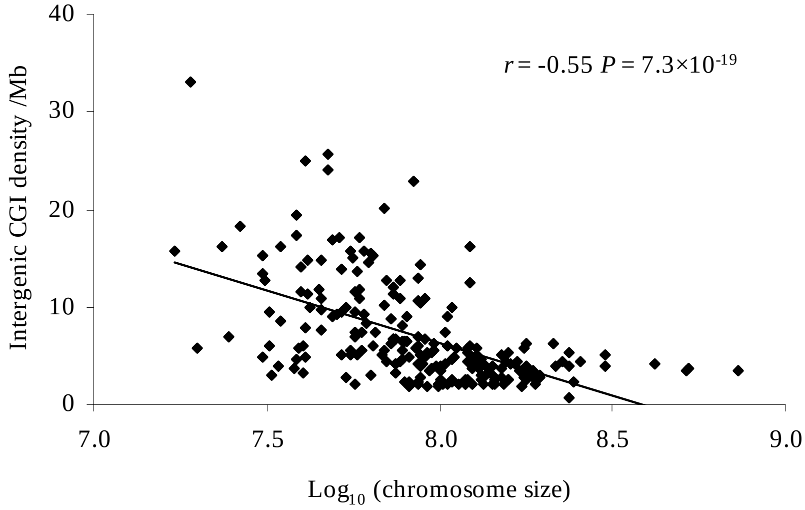

C

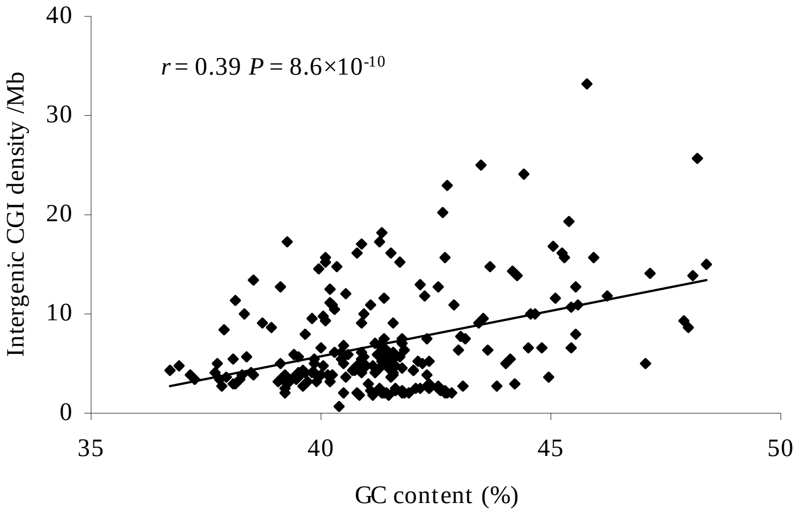
r = 0.39 P = 8.6×10-10
Intergenic CGI density /Mb
GC content (%)
0
10
20
30
40
35
40
45
50

D

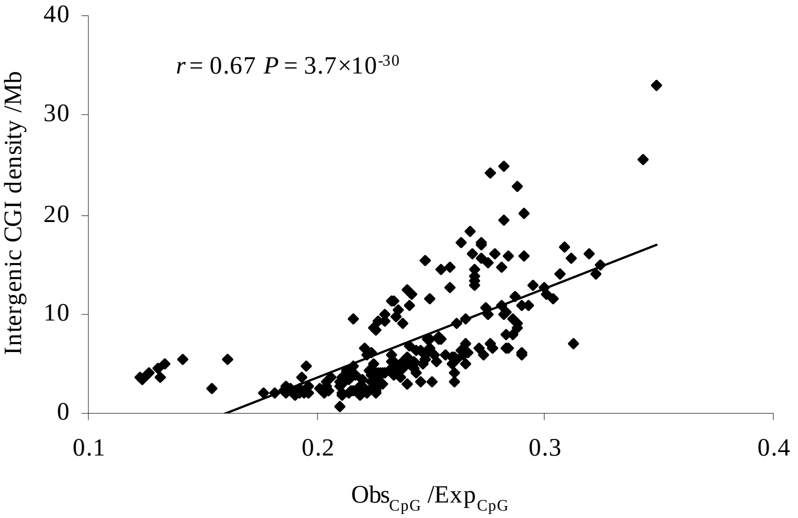
r = 0.67 P = 3.7×10-30
Intergenic CGI density /Mb
ObsCpG /ExpCpG
0
10
20
30
40
0.1
0.2
0.3
0.4
