# Supplementary material for: CpG island density and its correlations with genomic features in mammalian genomes
Source: Genome Biol. 2008 May 13;9(5):R79. doi: 10.1186/gb-2008-9-5-r79 (PMC2441465; doi:10.1186/gb-2008-9-5-r79)
Supplement: Additional file 4 — Correlations between CGI density and average recombination rate (cM/Mb) in the human, mouse and rat genomes. [file gb-2008-9-5-r79-S4.pdf]

Figure S3 CGI density (per Mb) vs. recombination rate (cM/Mb) in (**A**) humans (1-Mb window), **(B)** humans (10-Mb window), **(C)** mice (5-Mb window), **(D)** mice (10-Mb window), **(E)** rats (5-Mb window), and **(F)** rats (10-Mb window). The correlation coefficients are summarized in Table 3. The data in **A** were based on the deCODE genetic map and downloaded from the UCSC Genome Browser. The data in **B-F** were prepared by Jensen-Seaman et al. (2004).

A

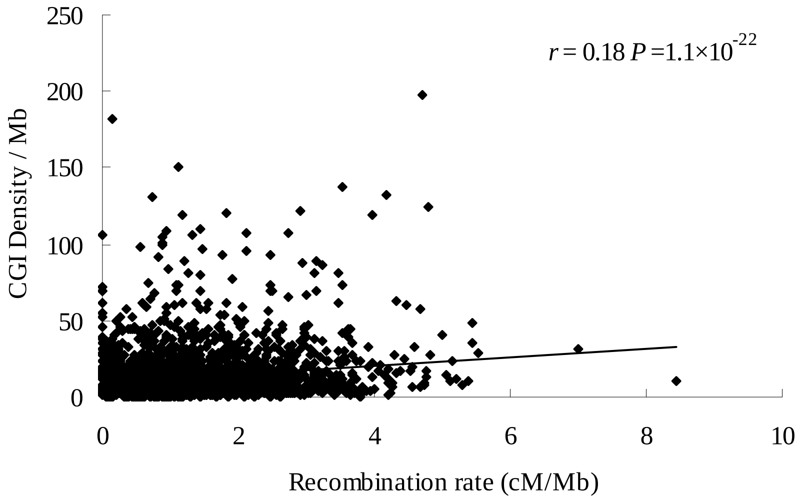

B

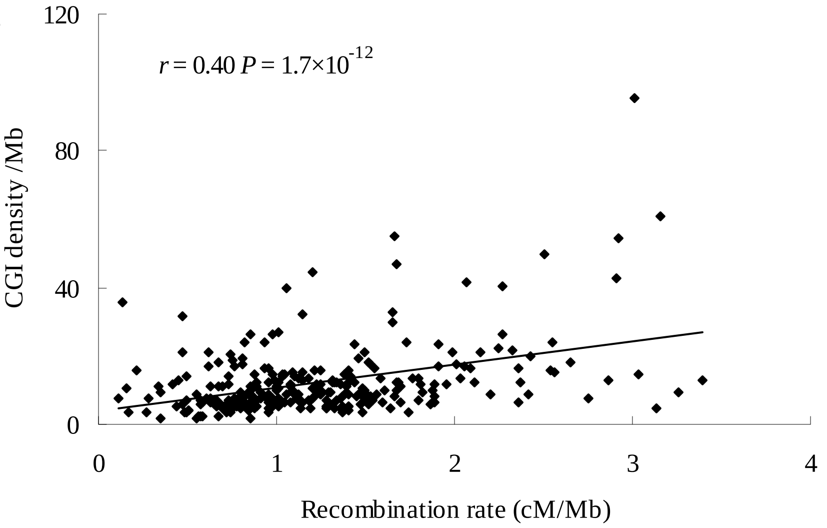

C

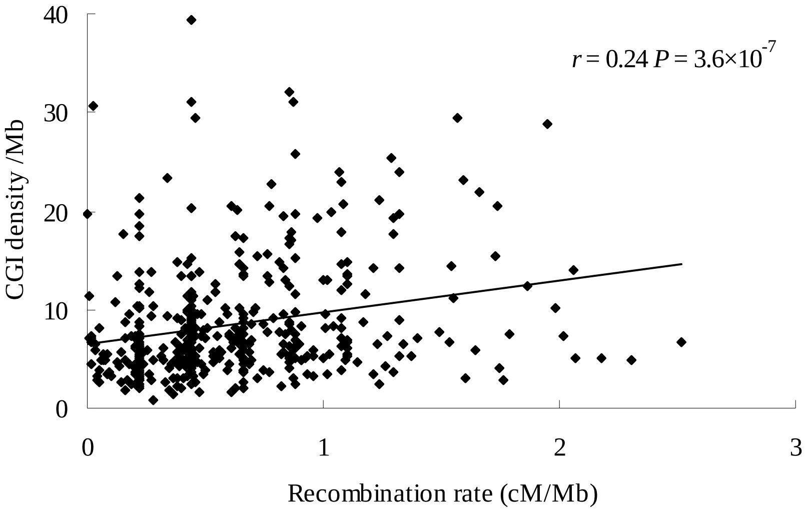
40
30
20
10
0
CGI density /Mb
r = 0.24 P = 3.6×10-7
0
1
2
3
Recombination rate (cM/Mb)

D

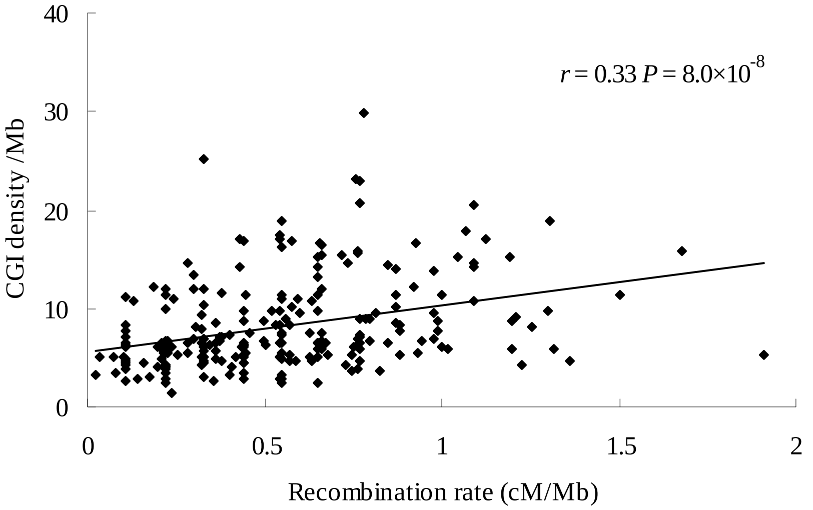
40
30
20
10
0
CGI density /Mb
r = 0.33 P = 8.0×10-8
0
0.5
1
1.5
2
Recombination rate (cM/Mb)

E

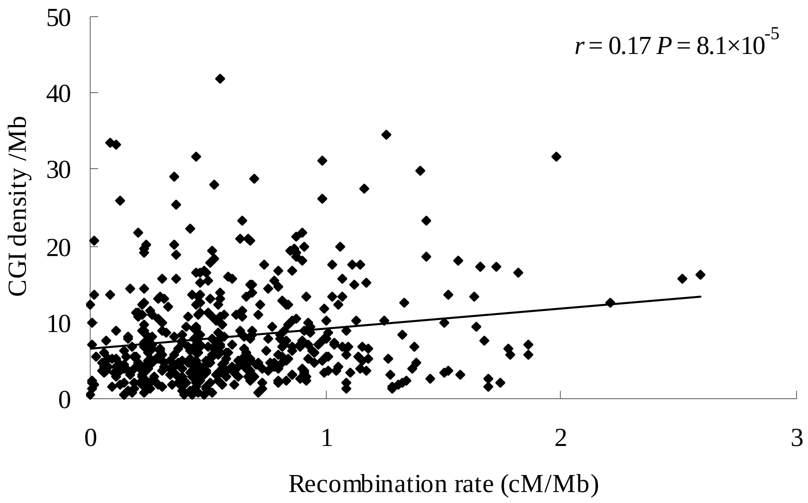

50
40
30
20
10
0
CGI density /Mb
r = 0.17 P = 8.1×10-5
0
1
2
3
Recombination rate (cM/Mb)

F

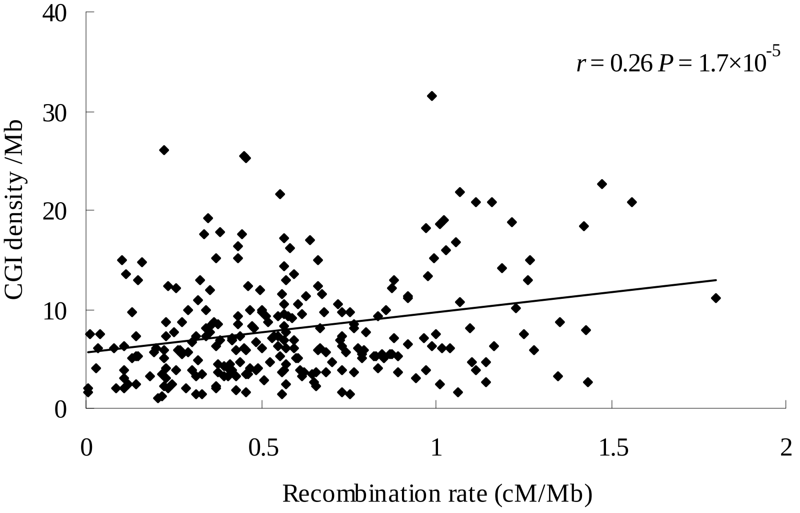

40
30
20
10
0
CGI density /Mb
r = 0.26 P = 1.7×10-5
0
0.5
1
1.5
2
Recombination rate (cM/Mb)
