# Supplementary material for: CpG island density and its correlations with genomic features in mammalian genomes
Source: Genome Biol. 2008 May 13;9(5):R79. doi: 10.1186/gb-2008-9-5-r79 (PMC2441465; doi:10.1186/gb-2008-9-5-r79)
Supplement: Additional file 5 — Comparison of CpG islands and other genomic features between mammalian and non-mammalian genomes. [file gb-2008-9-5-r79-S5.pdf]

Figure S4. Correlations between CGI density and genomic features in 9 mammalian genomes and 5 non-mammalian genomes. **(A-B)** CGI density (per Mb) vs. $\log_{10}$(chromosome size). **(C-D)** CGI density (per Mb) vs. chromosome $\text{Obs}_{\text{CpG}}/\text{Exp}_{\text{CpG}}$.

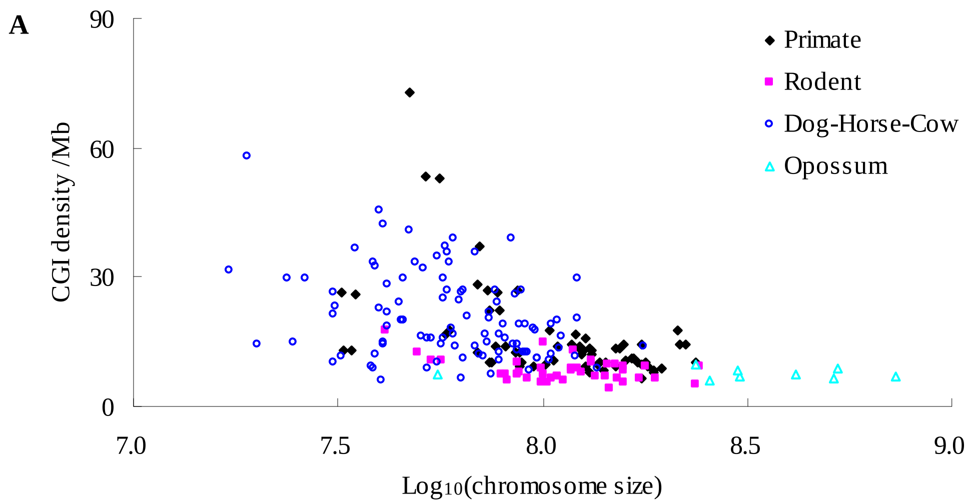

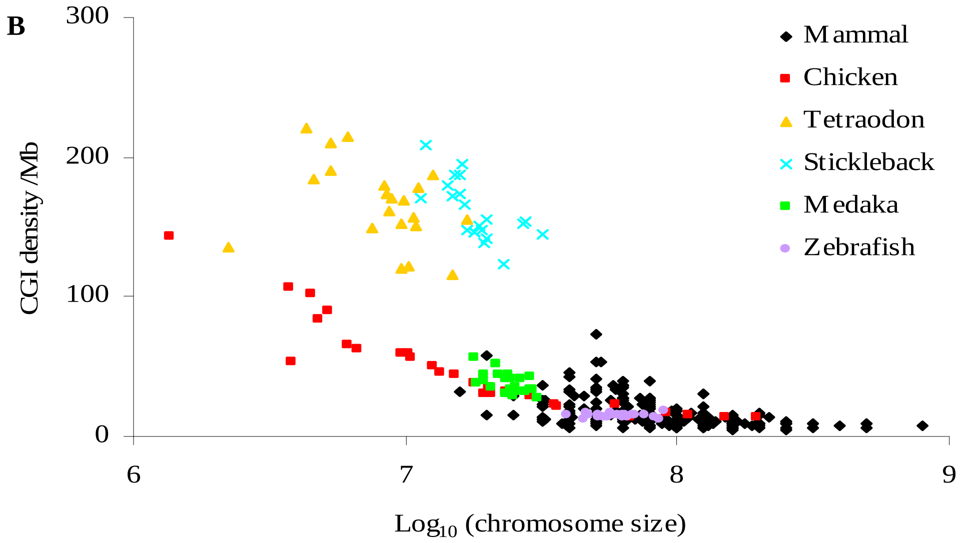

C

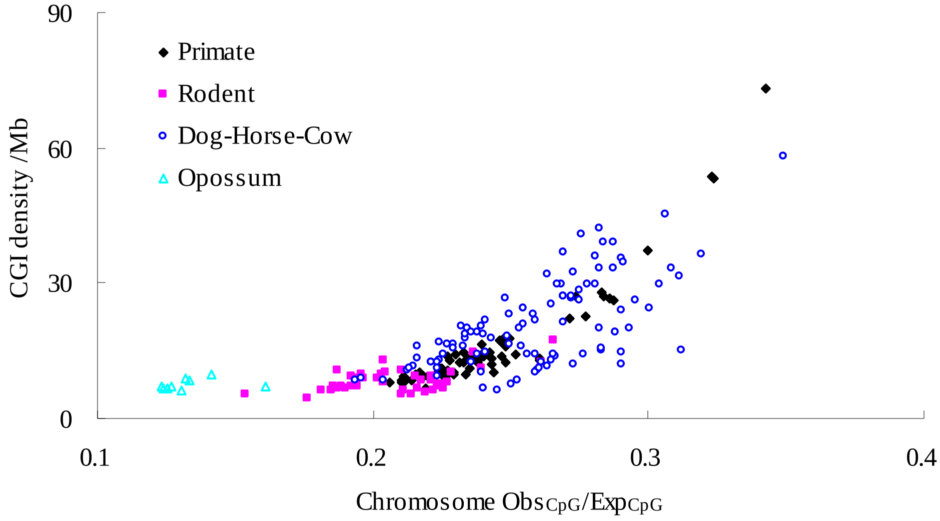

90
60
30
0
CGI density /Mb
Primate
Rodent
Dog-Horse-Cow
Opossum
0.1
0.2
0.3
0.4
Chromosome Obs CpG/Exp CpG

D

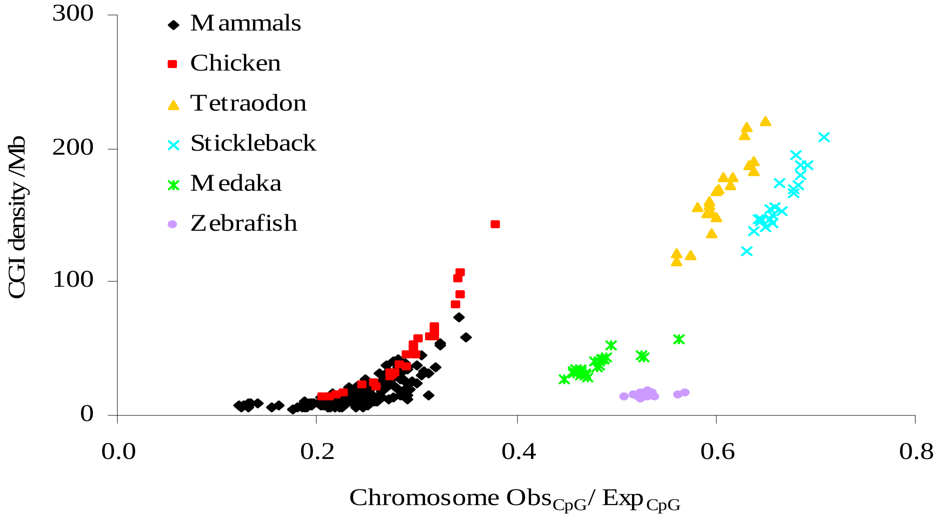

300
200
100
0
CGI density /Mb
Mammals
Chicken
Tetraodon
Stickleback
Medaka
Zebrafish
0.0
0.2
0.4
0.6
0.8
Chromosome Obs CpG/ Exp CpG
