# Supplementary material for: CpG island density and its correlations with genomic features in mammalian genomes
Source: Genome Biol. 2008 May 13;9(5):R79. doi: 10.1186/gb-2008-9-5-r79 (PMC2441465; doi:10.1186/gb-2008-9-5-r79)
Supplement: Additional file 6 — Correlations between CGI density and genomic features in mammalian genomes using the Gardiner-Garden and Frommer algorithm in the non-repeat portions of genomes. In both Additional data files 6 and 7, the platypus chromosomes were excluded because of incomplete genome sequence data and chromosome data. The conclusion would be the same when the platypus data were included. [file gb-2008-9-5-r79-S6.pdf]

Figure S5 Correlations between CGI density and genomic features in 9 mammalian genomes. CGIs were identified by **Gardiner-Garden and Frommer**'s (1987) algorithm in the non-repeat portions of genomes. The platypus chromosomes were excluded because of incomplete genome sequence data and chromosome data. The conclusion would be the same when the platypus data were included. **(A)** CGI density (per Mb) vs. number of chromosome pairs. **(B)** CGI density (per Mb) vs. $\log_{10}$(chromosome size). **(C)** CGI density (per Mb) vs. chromosome GC content (%). **(D)** CGI density (per Mb) vs. chromosome Obs_{CpG}/Exp_{CpG}.

A

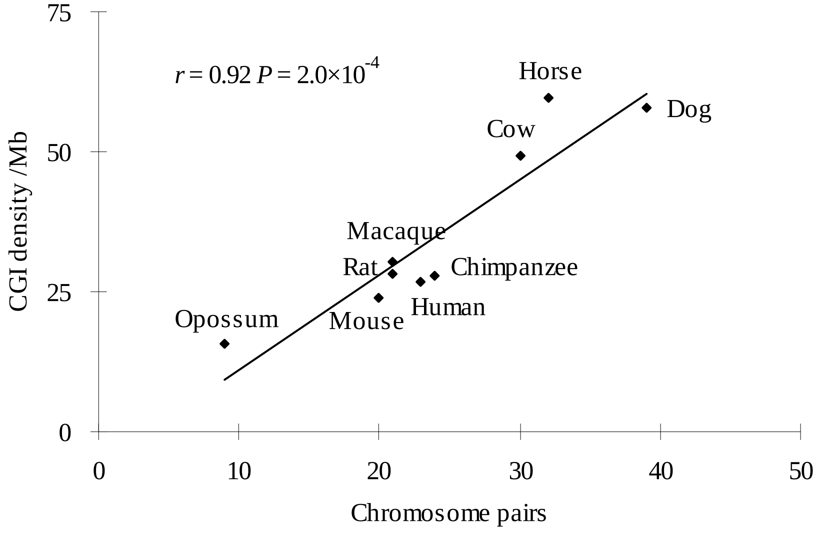

B

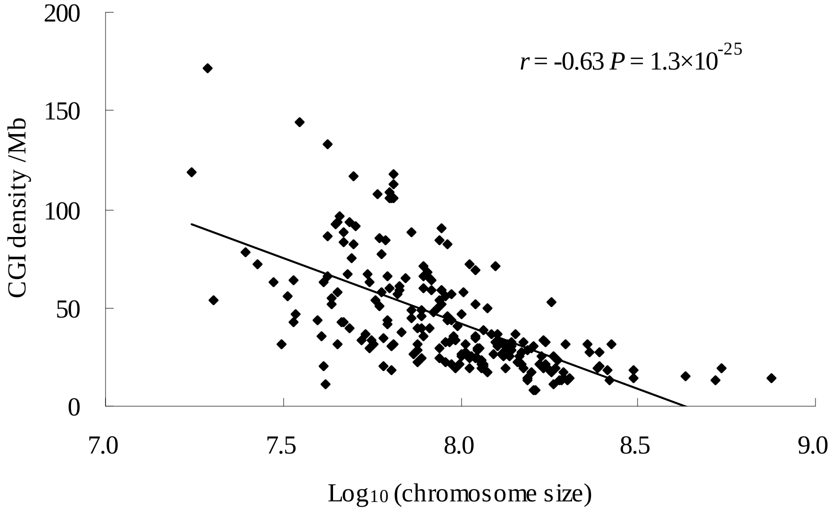

C

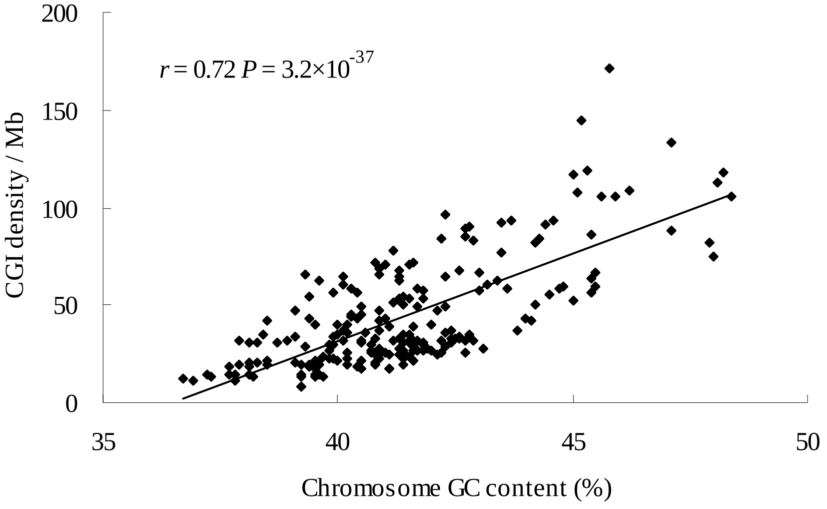

r = 0.72 P = 3.2×10-37
CGI density / Mb
200
150
100
50
0
35
40
45
50
Chromosome GC content (%)

D

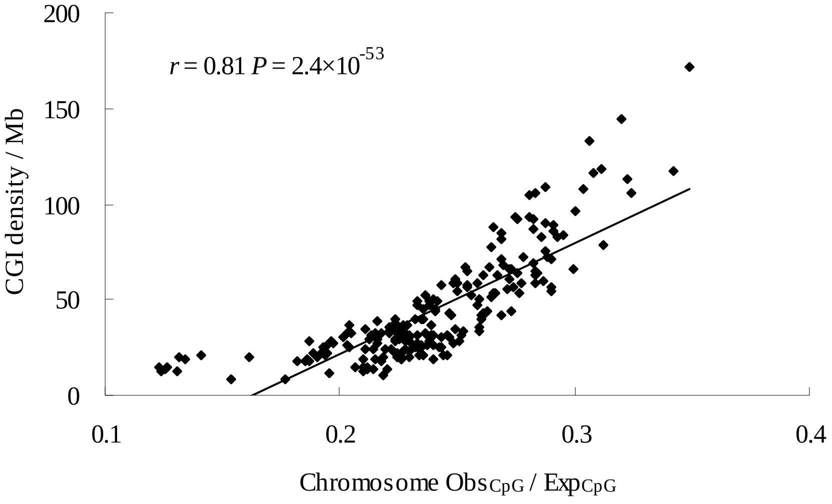

r = 0.81 P = 2.4×10-53
CGI density / Mb
200
150
100
50
0
0.1
0.2
0.3
0.4
Chromosome ObsCpG / ExpCpG
