# Supplementary material for: CpG island density and its correlations with genomic features in mammalian genomes
Source: Genome Biol. 2008 May 13;9(5):R79. doi: 10.1186/gb-2008-9-5-r79 (PMC2441465; doi:10.1186/gb-2008-9-5-r79)
Supplement: Additional file 7 — Correlations between CGI density and genomic features in mammalian genomes using the CpGcluster algorithm. In both Additional data files 6 and 7, the platypus chromosomes were excluded because of incomplete genome sequence data and chromosome data. The conclusion would be the same when the platypus data were included. [file gb-2008-9-5-r79-S7.pdf]

Figure S6. Correlations between CGI density and genomic features in 9 mammalian genomes. CGIs were identified by **CpGcluster** algorithm. The platypus chromosomes were excluded because of incomplete genome sequence data and chromosome data. The conclusion would be the same when the platypus data were included. **(A)** CGI density (per Mb) vs. number of chromosome pairs. **(B)** CGI density (per Mb) vs. $\log_{10}$(chromosome size). **(C)** CGI density (per Mb) vs. chromosome GC content (%). **(D)** CGI density (per Mb) vs. chromosome Obs_{CpG}/Exp_{CpG}.

A

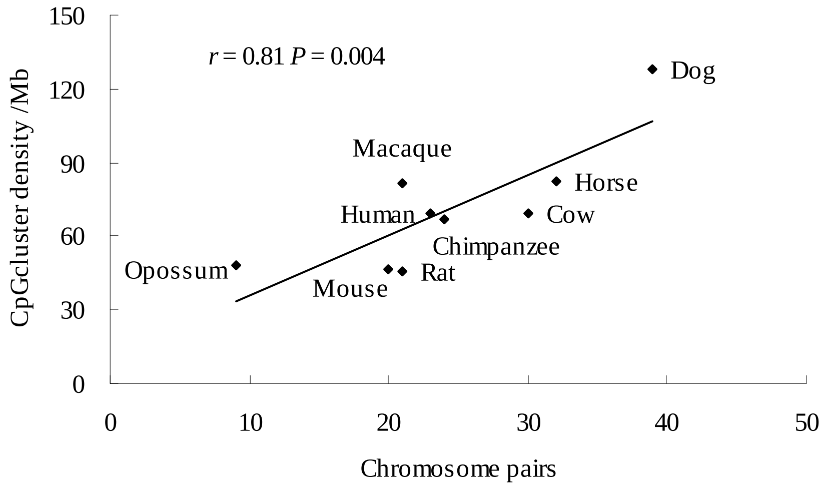

B

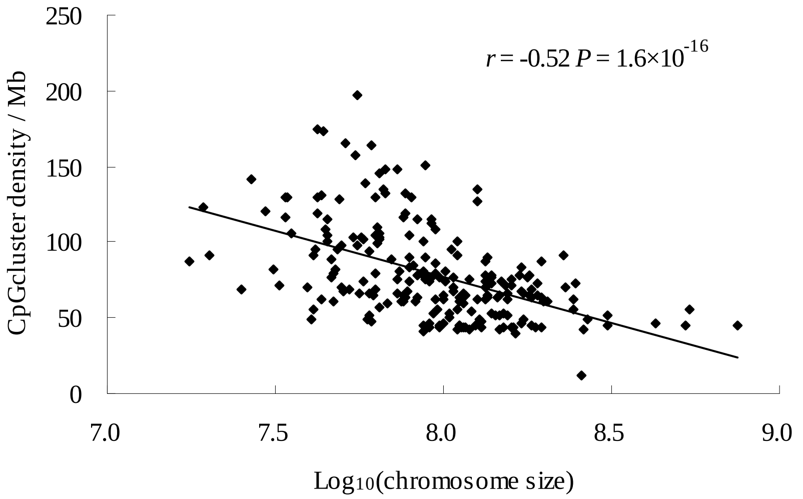

C

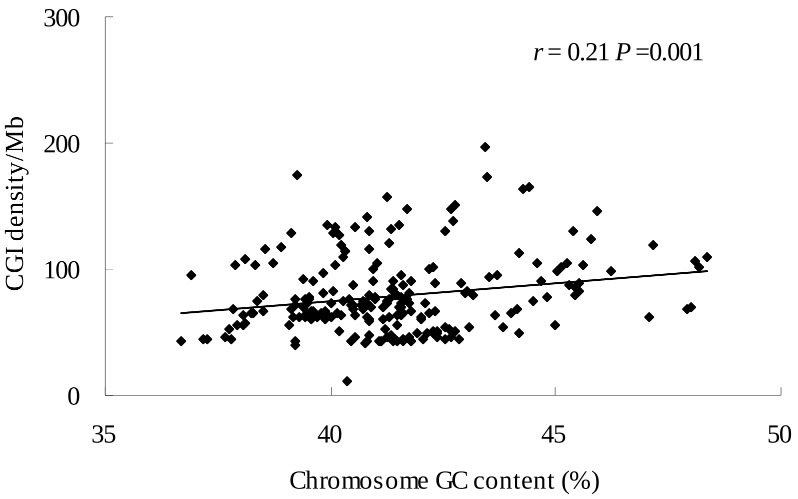

r = 0.21 P =0.001
300
200
100
0
35
40
45
50
CGI density/Mb
Chromosome GC content (%)

D

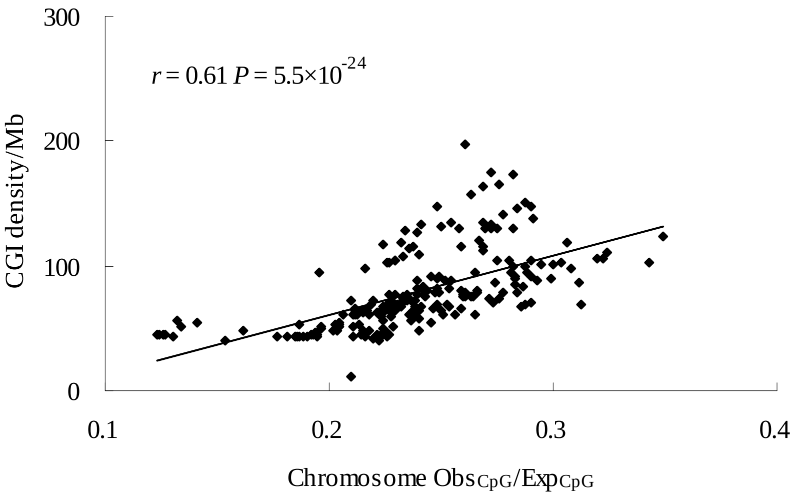

r = 0.61 P = 5.5×10^-24
300
200
100
0
0.1
0.2
0.3
0.4
CGI density/Mb
Chromosome ObsCpG/ExpCpG
